# PD-L1 Expression with Epithelial Mesenchymal Transition of Circulating Tumor Cells Is Associated with Poor Survival in Curatively Resected Non-Small Cell Lung Cancer

**DOI:** 10.3390/cancers11060806

**Published:** 2019-06-11

**Authors:** Yariswamy Manjunath, Sathisha V. Upparahalli, Diego M. Avella, Chelsea B. Deroche, Eric T. Kimchi, Kevin F. Staveley-O’Carroll, Charles J. Smith, Guangfu Li, Jussuf T. Kaifi

**Affiliations:** 1 Department of Surgery; Ellis Fischel Cancer Center, University of Missouri, One Hospital Drive, Columbia, MO 65212, USA; yariswamym@health.missouri.edu (Y.M.); venkateshaiahs@health.missouri.edu (S.V.U.); avellapatinod@health.missouri.edu (D.M.A.); kimchie@health.missouri.edu (E.T.K.); ocarrollk@health.missouri.edu (K.F.S.-O.); liguan@health.missouri.edu (G.L.); 2 Harry S. Truman Memorial Veterans’ Hospital, Columbia, MO 65212, USA; smithcj@health.missouri.edu; 3 Health and Medical Informatics/Office of Medical Research; Ellis Fischel Cancer Center, University of Missouri, One Hospital Drive, Columbia, MO 65212, USA; derochec@health.missouri.edu; 4 Department of Radiology; Ellis Fischel Cancer Center, University of Missouri, One Hospital Drive, Columbia, MO 65212, USA

**Keywords:** PD-L1, epithelial-mesenchymal transition, circulating tumor cells, liquid biomarkers, non-small cell lung cancer

## Abstract

In addition to the FDA-approved definition of a circulating tumor cell (CTC), various CTC phenotypes have been discovered. Epithelial-mesenchymal transition (EMT) of cancer cells is directly linked to PD-L1 upregulation. The goal of the study was to investigate PD-L1 expression and EMT in CTCs of non-small cell lung cancer (NSCLC) patients, and perform an outcome analysis. Prospectively, 7.5 mL peripheral blood was collected from 30 NSCLC patients that underwent surgery and 15 healthy controls. CTCs were enriched by size-based microfilter and immunofluorescence stainings performed (cytokeratin (CK) 8/18/19, EpCAM, CD45, PD-L1, EMT markers vimentin, and N-Cadherin, DAPI). Patient-matched NSCLC tissues were also stained. CTC staining intensity was quantified with a software and correlated with patient-matched NSCLC tissues and survival. PD-L1 and EMT markers were expressed at significantly higher proportions in CTCs than patient-matched NSCLC tissues (*p* < 0.05); ≥3 PD-L1^pos^/EMT^pos^CTCs were associated with significantly poorer survival after curative surgery (*p* < 0.05). No CTCs were detected in 15 healthy controls. This study shows that PD-L1 expression and EMT of CTCs is a negative survival predictor for NSCLC patients. The therapeutic role of the molecular linkage of PD-L1 and EMT will need to be further investigated, as linked pathways could be targeted to improve NSCLC outcome.

## 1. Introduction

Lung cancer is by far the leading cause of cancer-related deaths, with non-small cell lung cancer (NSCLC) being the most common subtype. Circulating tumor cells (CTCs) have detached from a tumor and are found in the blood of cancer patients. After FDA clearance of the CellSearch^®^ system for CTC enumeration in 2007, CTCs are defined as cells that express cytokeratins (CKs) 8/18 and/or 19 and EpCAM (epithelial cell markers) and that do not express CD45 (a leukocyte marker), with a well-defined DAPI^pos^ nucleus [1]. However, this immunoaffinity technology results in an incomplete assessment of the total number of circulating cancer-associated cells in the blood [2,3,4]. Other potentially most relevant CTCs carry a stem cell, mesenchymal, and immune cell phenotype, and these remain undetected with many CTC isolation technologies [5]. Non-affinity, size-based microfilter techniques are not limited to specific surface markers, allowing additional phenotype analysis of CTCs by application of a simple and cost-effective method for quenching of fluors with borohydride of immunofluorescently stained CTCs, followed by sequential restaining for additional biomarkers [6,7,8,9].

Epithelial-mesenchymal transition (EMT) is considered to be a critical process for detachment of cancer cells from the primary tumor and gaining the ability to metastasize [10]. During EMT, EpCAM, E-Cadherin, and other epithelial markers are downregulated; conversely, mesenchymal markers, such as vimentin and N-Cadherin, are upregulated. In different solid cancers, we and other groups have identified CTCs that have undergone EMT [4,6,7,11]. Checkpoint inhibition of PD-1/PD-L1 interaction has proven to be a successful immunotherapeutic approach in metastatic lung cancer treatment [12,13]. PD-L1^pos^CTCs can be detected in cancer patients, and are associated with a poorer prognosis in NSCLC patients undergoing anti-PD-1 treatment [14,15]. There are data demonstrating that EMT of cancer cells is associated with PD-L1 upregulation, inducing immune tolerance towards cancer cells [16,17,18,19,20,21]. In NSCLC patients, EMT-CTCs have also been observed to co-express PD-L1 [22,23]. These findings led to our hypothesis that PD-L1^pos^/EMT^pos^CTCs are associated with poorer survival of NSCLC patients after curative surgery.

In a prospective trial of 30 surgically treated NSCLC patients, CTC and matched tumor tissue expression of PD-L1 and EMT markers vimentin and N-Cadherin was compared, and CTC phenotypes correlated with survival after curative treatment of NSCLC patients. Results suggest that PD-L1 is significantly upregulated in EMT-CTCs, and PD-L1^pos^/EMT^pos^CTCs are associated with poor survival of NSCLC patients.

## 2. Results

### 2.1. Characteristics of Patients, CTC Counts

**Figure 1:**
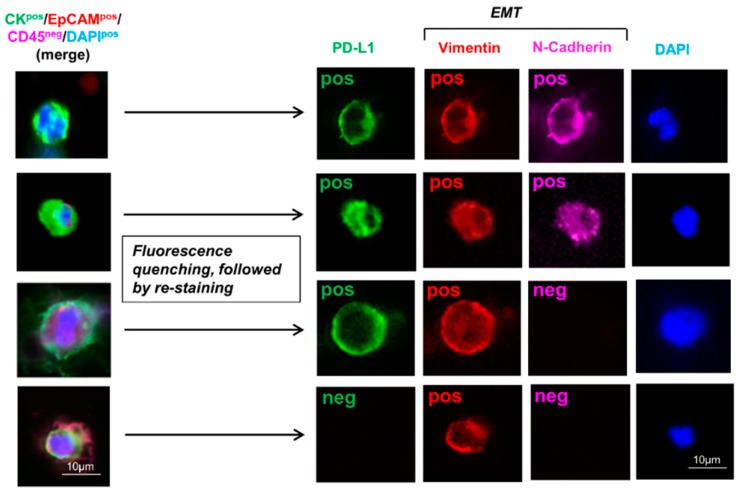
CK^pos^/EpCAM^pos^/CD45^neg^CTCs and CTC expression analysis for PD-L1, vimentin, and N-Cadherin in NSCLC patients. 7.5 mL blood was drawn, CTCs were enriched by microfilter isolation and immunofluorescence staining was performed for cytokeratins (CK) 8/18 and/or 19, EpCAM, CD45, and the nucleus identified with DAPI. Following identification of traditional CK^pos^/EpCAM^pos^/CD45^neg^CTCs (left panels showing merged images), fluorescence quenching with borohydride, followed by re-staining by immunofluorescence for checkpoint inhibitor target PD-L1 and epithelial-mesenchymal transition (EMT) markers vimentin and N-Cadherin was performed. Different CTC expression patterns with regard to PD-L1, vimentin, and N-Cadherin are shown.

**Figure 2:**
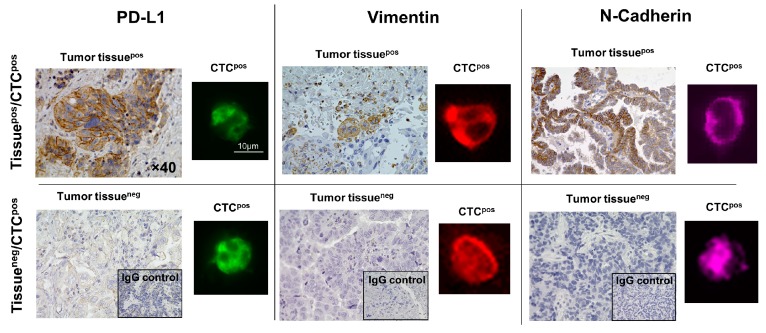
PD-L1 and EMT markers vimentin and N-Cadherin expressions determined by immunostaining in CTCs and patient-matched non-small cell lung cancer (NSCLC) tissues. Shown are representative images of expression patterns of immunohistochemically stained NSCLC tissues and patient-matched CTCs that were stained by immunofluorescence for PD-L1 and EMT markers.

**Figure 3:**
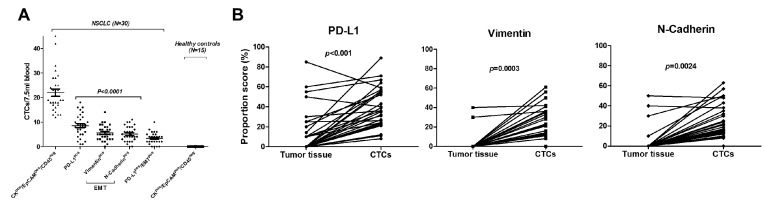
CTC counts, and comparative CTC expression and patient-matched tumor tissue analysis for PD-L1, vimentin, and N-Cadherin in NSCLC patients (*N* = 30). (**A**) Counts per 7.5 mL of blood of traditional CK^pos^/EpCAM^pos^/CD45^neg^CTCs are shown. CTC positive expression for PD-L1, vimentin, and N-Cadherin (defined as ≥50% mean intensity determined by quantification software) was determined after quenching of fluorescence and immunofluorescence re-staining with specific antibodies. PD-L1^pos^CTCs were detected at a significantly higher rate than vimentin^pos^CTCs and/or N-Cadherin^pos^CTCs (*p*-value was calculated by non-parametric Tukey’s multiple comparison analysis). EMT^pos^CTCs had to co-express both vimentin and N-Cadherin. PD-L1^pos^/EMT^pos^CTCs were observed in 26/30 (86.7%) of NSCLC patients. No CTCs were identified in 15 healthy control subjects. (**B**) Comparative analysis of NSCLC tumor tissues and patient-matched CTCs (*N* = 30) expression proportion (%) scores for PD-L1 (left panel), vimentin (middle panel), and N-Cadherin (right panel). PD-L1, vimentin, and N-Cadherin were statistically significantly higher expressed in CTCs than in patient-matched NSCLC tissues (*p*-values were calculated with non-parametric Wilcoxon signed-rank test for matched pairs).

In a prospective trial, 7.5 mL of whole blood was analyzed for CTC counts from 45 subjects. Out of these, 30 patients were diagnosed with NSCLC (all treatment-naïve) and underwent curative surgical resection, and 15 healthy controls were never-smokers (mean age 45.5; median age 43; range 30–65) (Table 1). NSCLC histologic subtypes and stages are listed in Table 1. Mean age of NSCLC patients was 65 years (range 50–79), and 14 (46.7%) were females. CTCs were enriched by a validated size-based isolation platform and in alignment with the FDA-approved definition of a CTC identified by immunostaining as CK^pos^/EpCAM^pos^/CD45^neg^ with a DAPI^pos^ nucleus (Figure 1). In 30 NSCLC patients, the mean CK^pos^/EpCAM^pos^/CD45^neg^CTC count was 21.97 (SEM ± 1.48) (median 19 (range 12–45)). As expected, there were significantly increasing CTC counts from stage I to III (*p* = 0.0006; Kruskal–Wallis test). Following immunofluorescent staining with these FDA-approved CTC criteria, quenching of fluors with borohydride was performed, followed by sequential restaining of the CTCs with additional biomarkers PD-L1, vimentin, and N-Cadherin (Figure 1 and Figure 2). PD-L1^pos^/EMT^pos^CTCs were identified at a lower, yet consistent, rate with a mean count of 3.37 (±0.42) (3 (0–10)) (Figure 3). Also, PD-L1^pos^/EMT^pos^CTCs counts significantly increased from stage I to stage II/III (*p* = 0.0292) (Table 1). No CTCs were identified in the 15 healthy control subjects. Following enumeration of these traditional CK^pos^/EpCAM^pos^/CD45^neg^CTCs, immunofluorescence quenching and expression analysis of these CTCs for checkpoint inhibitor target PD-L1 and EMT markers vimentin and N-Cadherin was performed by immunofluorescence staining (Figure 1). CTC expression for PD-L1, vimentin, and N-Cadherin was determined. Positivity was defined as ≥50% mean immunofluorescence intensity determined by quantification software, independent of membranous, nuclear, or cytoplasmic expression localization. PD-L1^pos^CTCs were found in all 30 (100%), Vimentin^pos^CTCs in 29/30 (96.7%) patients, and N-Cadherin^pos^CTCs in 28/30 (93.3%) NSCLC patients (Table 2).

EMT^pos^CTCs had to co-express both vimentin and N-Cadherin. PD-L1^pos^/EMT^pos^CTCs were observed in 26/30 (86.7%) NSCLC patients (Table 1; Figure 1 and Figure 3). As expected, both CK^pos^/EpCAM^pos^/CD45^neg^CTCs and PD-L1^pos^/EMT^pos^CTCs were identified at increasing levels from AJCC stages I to II/IIIA.

### 2.2. CTCs Express PD-L1 and EMT Markers Vimentin and N-Cadherin at Higher Rates than Matched Primary NSCLC Tissue

Patient-matched NSCLC tumor tissues (*n* = 30) were harvested at the time of surgical resection and stained for PD-L1, EMT markers vimentin, and N-Cadherin (Table 2; Figure 2, Figure 3). Positive expression of PD-L1 was noted in 14/30 (46.7%), whereas EMT markers were observed in lower frequencies: Vimentin in 2/30 (6.7%) and N-Cadherin in 4/30 (13.3%) of NSCLC tissues (Table 2). No NSCLC tumor tissue was found to be triple PD-L1^pos^/vimentin^pos^/N-Cadherin^pos^. Expression proportion scores (%) of PD-L1^pos^CTCs, vimentin^pos^CTCs, and N-Cadherin^pos^CTCs or tissue tumor cells of all CK^pos^/EpCAM^pos^/CD45^neg^CTCs or all tissue tumor cells were determined (Table 2). Consistently, CTCs had a statistically significantly higher expression proportion score (%) than the matched primary NSCLC tissue (CTCs versus NSCLC tumor tissue: PD-L1: mean 39.20 (±3.72); median 36 (range 8–89) vs. 13.47 (±4.02); 0 (0–85) (*p* < 0.0001; non-parametric Wilcoxon signed-rank test for matched pairs); vimentin: 26.77 (±2.77); 23 (0–61) vs. 2.33 (± 1.64); 0 (0–40) (*p* = 0.0003); N-Cadherin: 24.47 (±3.04); 20 (0–63) vs. 4.33 (±2.28); 0 (0–50) (*p* = 0.0024)) (Figure 3). These data indicate that NSCLC primary tumor cells undergo EMT and upregulate PD-L1 once they detach from the primary tumor and enter the blood to become CTCs. 

### 2.3. Presence of ≥3 PD-L1^pos^/EMT^pos^CTCs Correlates with Outcome of Surgically Treated NSCLC Patients

**Figure 4:**
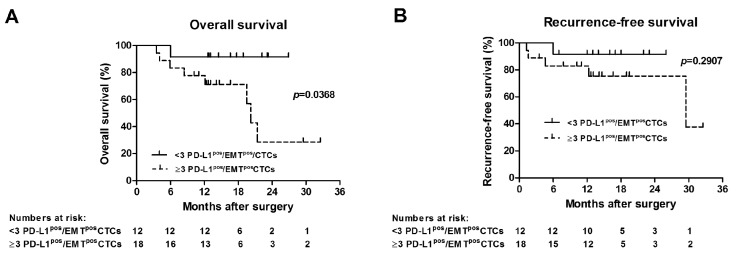
Presence of PD-L1^pos^/EMT^pos^CTCs is associated with shorter overall survival of NSCLC patients that underwent curative surgery. (**A**) Overall and (**B**) recurrence-free Kaplan–Meier survival curves of NSCLC (AJCC stage I–IIIA) patients (*N* = 30) that underwent curative lung surgery are presented. (**A**) Presence of ≥3 PD-L1^pos^/EMT^pos^CTCs was associated with significantly worse overall survival, as determined by univariate analysis (*p* = 0.0368; log-rank test). (**B**) Presence of ≥3 PD-L1^pos^/EMT^pos^CTCs was associated with more events of recurrences (5/18 (27.8%) versus 1/12 (8.3%)), although it did not reach level of significance (*p* = 0.2907, log-rank test; *p* = 0.3575, Fisher’s exact test).

All 30 NSCLC patients included for CTC analysis underwent complete surgical resections with curative intent for AJCC stages I–IIIA (Table 1). Median follow-up time after surgery was 14.3 months (range 3.5–32.5 months). Overall and recurrence-free survival was calculated from the time of surgical resection, and correlated with presence of PD-L1^pos^/EMT^pos^CTCs in 7.5 mL of blood before surgery. Presence of ≥3 PD-L1^pos^/EMT^pos^CTCs had a significant correlation with poorer overall survival, as determined by Kaplan–Meier survival analysis (*p* = 0.0368; log-rank test) (Figure 4A). Median overall survival of patients with ≥3 PD-L1^pos^/EMT^pos^CTCs was 20.2 months. Presence of ≥3 PD-L1^pos^/EMT^pos^CTCs was associated with more events of recurrences (5/18 (27.8%)) versus <3 PD-L1^pos^/EMT^pos^CTCs (1/12 (8.3%) recurrences), although it did not reach level of significance by univariate Kaplan–Meier survival (*p* = 0.2907; log-rank test) or contingency analysis (*p* = 0.3575; Fisher’s exact test), most likely due to small sample size and few number of events during the observation period (Figure 4B). Median recurrence-free survival of patients with ≥3 PD-L1^pos^/EMT^pos^CTCs was 29.5 months. In summary, presence of ≥3 PD-L1^pos^/EMT^pos^CTCs is associated with poorer overall survival of NSCLC patients that undergo curative surgical resection.

## 3. Discussion

Despite successful implementation of promising novel targeted agents for NSCLC therapy, overall five-year survival of lung cancer remains at approximately 18% only [24]. After FDA clearance of the CellSearch^®^ system for CTC detection in 2007, CTCs were traditionally defined as cells that are CK^pos^/EpCAM^pos^/CD45^neg^ with a DAPI^pos^ nucleus [1]. However, these criteria may not include CTCs that have undergone EMT [2,3,4]. In a prospective trial of 30 NSCLC patients that underwent curative lung surgery, we demonstrated that PD-L1 and EMT markers vimentin and N-Cadherin are upregulated in CTCs in comparison to tumor tissue, and that ≥3 PD-L1^pos^/EMT^pos^CTCs in 7.5 mL blood drawn before curative surgery are associated with poor overall survival. 

In the present study, we applied a validated microfilter CTC isolation method that has proven to provide consistent CTC detection results in our own and other investigator’s studies [25,26,27]. As initially described by Adams and colleagues, this microfilter analysis allows quenching of fluors, and restaining of CTCs for additional biomarkers using a cost-effective and straightforward protocol with borohydride [9]. CTCs have very heterogeneous pheno- and genotypes, with certain CTC subgroups having more metastatic potential than others [3]. EMT is a critical metastatic phenotype change required for detachment of cancer cells from the primary epithelial tumor [10,28]. During EMT, mesenchymal markers, such as vimentin, N-Cadherin, SNAIL, fibronectin, or β-catenin are upregulated. In different solid cancers, we and other groups have identified CTCs that carry an EMT phenotype [6,7,29,30]. Presence of ≥5 EMT-CTCs is associated with progressive disease in metastatic colorectal cancer patients [11]. Checkpoint inhibition of PD-1/PD-L1 interaction has proven to be a successful immunotherapeutic approach in metastatic lung cancer treatment [12,13]. In a seminal study, CTC PD-L1 expression was initially described in breast cancer patients [15]. Anti-PD-1 treatment responses in NSCLC are also noted if tumor tissues lack PD-L1 expression, and our present and other recent studies have confirmed that PD-L1 expression is higher in CTCs than in tumor tissue [31,32]. Also, pre-treatment PD-L1^pos^CTCs are associated with a poorer prognosis in NSCLC patients undergoing anti-PD-1 therapy with nivolumab [14]. These findings suggest that PD-L1 upregulation in CTCs leads to a survival benefit of metastatic cells [15]. Unsurprisingly, the response to PD-1 inhibitor therapy correlates with a reduction in PD-L1^pos^CTC [33,34].

It has been demonstrated that EMT is associated with increased expression of multiple immune checkpoints leading to immune tolerance [19,20]. Induction of EMT in epithelial cells leads to a PI3K/AKT pathway-dependent PD-L1 upregulation [16]. PD-L1-mediated CD8^pos^ tumor-infiltrating lymphocyte immunosuppression and subsequent metastasis is inhibited by EMT suppressing microRNA-200 [21]. NSCLC tissue analyses demonstrated association of PD-L1 expression with an EMT phenotype [18,35]. In our NSCLC cohort, we have now confirmed other investigators’ findings that CTCs coexpress PD-L1 and EMT markers vimentin and N-Cadherin as a possible mechanism for immune escape [23]. As a novelty, our study shows a significant association of PD-L1^pos^/EMT^pos^CTCs with NSCLC overall survival after curative surgical treatment. As it has been described by other investigators, we also observed that cellular PD-L1, vimentin, and N-Cadherin expression patterns in CTCs is differential (membranous, cytoplasmic, nuclear) [23,29,36,37,38]. In our analysis, PD-L1 and EMT marker positivity was defined independent from membranous, cytoplasmic, and nuclear localization. But in a recent publication, it was reported that PD-L1 expressed in the nucleus of vimentin-positive CTCs predicts poor prognosis in colorectal and prostate cancer patients [22]. The significance of differential cellular expression has to be further clarified, but investigators suggested that nuclear translocation of PD-L1 might cause resistance to T cell induced cytotoxicity and inhibit drug-induced apoptosis [36,39]. Future mechanistic studies will have to shed further light on the biology of these EMT and immune checkpoint interactions on a molecular level.

## 4. Materials and Methods

### 4.1. Patient Selection Criteria

The study was conducted in accordance with the Declaration of Helsinki, and the protocol was approved by the Ethics Committee of the University of Missouri and Truman VA Hospital (Institutional Review Board (IRB) approval numbers: IRB2010166, IRB2004401-VA). All subjects gave their informed consent for inclusion before they participated in the study. Trials were registered at *ClinicalTrials.gov* (NCT02838836/NCT03551951). Recruitment timeframe was from July 2016 to May 2018. Thirty NSCLC patients that underwent surgery for AJCC stage I–IIIA were prospectively included for analysis. Fifteen healthy never-smokers were included as negative controls.

### 4.2. Clinicopathological Data

Data were collected by reviewing electronic records, including imaging results and other relevant medical findings. The staging manual of the American Joint Committee on Cancer (AJCC), 8th Edition, was used. All cases were reviewed in multidisciplinary thoracic oncology conferences. Clinical follow-up data were obtained. Survival and recurrence data were gathered by reviewing the hospital records, direct communication with the treating physicians, and from the Cancer Registry of the State of Missouri. Overall survival was calculated from the date of surgical excision of the tumor to the date of death or last follow-up. NSCLC recurrences (loco-regional or distant metastases) were calculated from the date of surgical excision of the tumor to the date of diagnosis of the recurrences.

### 4.3. CTC Detection and Staining PD-L1 and EMT Markers Vimentin and N-Cadherin

Blood draws were done at the initial patient encounters in the outpatient clinics via peripheral vein phlebotomy before biopsies or surgeries were performed to avoid CTC spillage by tissue manipulation. The first 6 mL blood were discarded to avoid epithelial contamination from skin puncture. Then, 7.5 mL of blood were collected in CellSave^®^ tubes (Menarini-Silicon Biosystems, Huntingdon Valley, PA, USA) (as recommended by the manufacturer of CellSieve^™^ microfilters (Creatv MicroTech, Rockville, MD, USA)). Within 1 hour, CTCs were enriched by size with an established microfilter platform that has been successfully used by other investigators and our group [25,27,40,41]. Blood was passed through filters with a syringe pump (KD Scientific Legato 110 CMT, Analytical West Instruments, CA, USA). Enriched cells that were adherent to the filter were characterized by immunofluorescence staining on the filter with an antibody cocktail (CK8/18/19-FITC, EpCAM-PE, CD45-Cy5). Microfilters were then transferred on a microscopic slide, anti-fade mounting medium with DAPI (Cell Signaling Technology, Danvers, MA, USA) added, a coverslip placed on the filter, and incubated in the dark at room temperature for 8 hours. In alignment with the FDA-approved definition of a CTC, we defined a traditional CTC as CK^pos^/EpCAM^pos^/CD45^neg^ with a DAPI^pos^ nucleus inside the cytoskeleton [1]. Immunostainings were standardized using human NSCLC cell line A549 (ATCC^®^ CCL-185™) that were spiked into whole blood of healthy volunteers.

For CTC PD-L1, vimentin, and N-Cadherin expression analysis, previously stained filters underwent fluorescence quenching with a simple, cost-effective, and established protocol using sodium borohydride [9]. This protocol allows multi-phenotype analysis of CTCs using sequential fluorescent quenching and restaining for additional biomarkers. Following quenching of immunofluorescence staining for CK/EpCAM/CD45, immunofluorescence restaining with an antibody cocktail containing anti-PD-L1 Alexa Fluor 488 (rabbit mAb; clone D8T4X; Cell Signaling), anti-vimentin Alexa 555 (rabbit mAb; clone D21H3; Cell Signaling), anti-N-Cadherin Alexa 647 (rabbit mAb; clone EPR1791-4; abcam), and DAPI was performed. PD-L1, vimentin, and N-Cadherin expression was analyzed on CTCs that had undergone quenching and restaining, but were previously identified as CK8/18/19^pos^/EpCAM^pos^/CD45^neg^ with a DAPI^pos^ nucleus. As recently described by other investigators, we also observed differential membranous, cytoplasmic, and/or nuclear expression patterns of PD-L1, vimentin, and N-Cadherin [23,29,36,37,38]. The MetaMorph Microscopy Image Analysis Software (v.7.8.12) (Molecular Devices, San Jose, CA, USA) was used for fluorescence intensity quantification of the CTCs that were re-stained for PD-L1, vimentin, and N-Cadherin. CTC expression positivity was defined as ≥50% mean immunofluorescence intensity of the mean fluorescence intensity of all CTCs analyzed, independent of membranous, nuclear, or cytoplasmic expression localization. Proportions (in percentages) of PD-L1/vimentin/N-Cadherin^pos^CTCs of all CK^pos^/EpCAM^pos^/CD45^neg^CTCs were calculated. CTCs were defined as EMT^pos^ if they co-expressed both vimentin and N-Cadherin. Images were taken using Zeiss Axiovert 200M inverted microscope (Oberkochen, Germany) equipped with a Hamamatsu ORCA-ER CCD (Shizuoka, Japan) camera. All analyses were performed blinded to reduce observer bias.

### 4.4. NSCLC Tissue Immunostaining

Patient-matched NSCLC tumor tissues and adjacent normal lung tissue were snap-frozen in liquid nitrogen and stored at −70 °C. Tissue pieces were then placed in OCT Embedding Compound, and 5 µm thick sections were cut with a cryostat. Presence of tumor was confirmed after hematoxylin and eosin (H&E) staining by a pathologist. Slides were treated with citrate-based antigen unmasking solution (Vector Laboratories, Burlingame, CA, USA). Sections were blocked non-specific proteins with horse serum, and treated with BLOXALL Endogenous Peroxidase Blocking Solution (Vector). Anti-PD-L1 antibody (rabbit mAb; clone 73-10; abcam, Cambridge, MA, USA), anti-vimentin (mouse mAb; clone V9; abcam), and anti-N Cadherin (mouse mAb; clone 5D5; abcam) were incubated at 4 °C overnight. Rabbit or mouse IgG (Santa Cruz Biotechnology, Dallas, TX, USA) were used as isotype controls. ImmPRESS™ HRP Anti-Rabbit/-Mouse IgG Peroxidase Polymer Detection Kit (Vector) were used as secondary antibodies, ImmPACT DAB Peroxidase (HRP) substrate for detection, and hematoxylin (Vector) as counterstain. PD-L1, vimentin, and N-Cadherin expression were determined by using a tumor proportion score as applied in the diagnostic clinical setting: Percentage of tumor cells showing partial or complete membranous, cytoskeletal, and/or nuclear staining for PD-L1, vimentin, and N-Cadherin [13]. Normal lung tissues were stained for PD-L1, vimentin, and N-Cadherin as controls, and expected patterns of expression were observed [42].

### 4.5. Statistical Analysis

For this study, key elements of the prospective-specimen-collection, retrospective-blinded-evaluation (PRoBE) design were applied [43]. With this methodology, specimens and clinical data were collected prospectively, with the analytic personnel being without knowledge of the outcome (blinded). Statistical tests performed were the non-parametric Tukey’s multiple comparison analysis, Mann Whitney test, Friedman test, non-parametric Wilcoxon signed-rank test for matched pairs, and Fisher’s exact test. Survival was analyzed by the Kaplan–Meier curve method, and the log-rank test was used for univariate analysis. Significance statements refer to a *p*-value of <0.05. Statistical analyses were performed using software Prism version 5.00 (GraphPad, La Jolla, CA, USA).

## 5. Conclusions

In summary, data from this study support recent findings that malignant progression is driven by EMT and PD-L1, and is associated with poorer survival of NSCLC patients. These observations might open novel avenues in liquid biopsy profiling to select NSCLC patients for immunotherapies or even combinational treatments targeting EMT and the PD-1/PD-L1 axis. In hope of future improvement of currently disappointing NSCLC outcomes, molecular studies may clarify the underlying relationship of EMT and immune tolerance towards cancer cells.

## Figures and Tables

**Table 1:** Subjects’ characteristics and analysis for circulating tumor cells (CTCs).

Characteristics	*N*	CK^pos^/EpCAM^pos^/CD45^neg^CTCs: Detected in *N* (%) Mean (±SEM); Median (Range)	PD-L1^pos^/EMT^pos^CTCs: Detected in *N* (%) Mean (±SEM); Median (Range)
Total # of subjects	45		
NSCLC (all treatment-naïve)	30	30 (100%) 21.97 (±1.48); 19 (12–45)	26 (86.7%) 3.37 (±0.42); 3 (0–10)
Age (median/range)	65 (50–79)		
Gender			
Females (%)	14 (46.7%)		
Males (%)	16 (53.3%)		
Histologic subtype			
Adenocarcinoma	18 (60%)		
Squamous cell	10 (33.3%)		
Large-cell neuroendocrine	2 (18.7%)		
AJCC stages (8th ed.)			
I	16 (53.3%)	17.25 (±1.09); 17 (12–28)	2.44 (±0.46); 2 (0–6)
II	8 (26.7%)	23.75 (±1.79); 24 (18–33)	5.13 (±1.03); 5 (0–10)
IIIA	6 (20%)	32.17 (±3.83); 30 (23–45)	3.50 (±0.43); 4 (2–5)
		(*p* = 0.0006) *	(*p* = 0.0292)
Healthy controls	15	0	0
Age (median/range)	43 (30–65)		

*p*-values were determined with Kruskal–Wallis test; Abbreviations: NSCLC: Non-small cell lung cancer; CTCs: Circulating tumor cells; SEM: Standard error of the mean. AJCC: American Joint Committee on Cancer.

**Table 2:** Expression proportion scores of patient-matched CTCs and NSCLC tumor tissues (*N* = 30).

Markers Analyzed	CTCs Positive in *N* (%) CTC Proportion Scores (%): Mean (±SEM); Median (Range)	Tissue Positive in *N* (%) Tumor Proportion Scores (%): Mean (±SEM); Median (Range)	*p*-Value
PD-L1^pos^	30 (100%)	14 (46.7%)	
	39.20 (±3.72); 36 (8–89)	13.47 (±4.02); 0 (0–85)	<0.0001
Vimentin^pos^	29 (96.7%)	2 (6.7%)	
	26.77 (±2.77); 23 (0–61)	2.33 (±1.64); 0 (0–40)	0.0003
N-Cadherin^pos^	28 (93.3%)	4 (13.3%)	
	24.47 (±3.04); 20 (0–63)	4.33 (±2.28); 0 (0–50)	0.0024
PD-L1^pos^/EMT^pos^	26 (86.7%)	0	
	15.70 (±1.97); 15 (0–43)	0	<0.0001

Abbreviations: NSCLC: Non-small cell lung cancer; CTCs: Circulating tumor cells; SEM: Standard error of the mean; *p*-values were determined with non-parametric Wilcoxon signed-rank test for matched pairs.

## References

[1] Racila E., Euhus D., Weiss A.J., Rao C., McConnell J., Terstappen L.W., Uhr J.W. (1998). Detection and characterization of carcinoma cells in the blood. Proc. Natl. Acad. Sci. USA.

[2] de Wit S., van Dalum G., Lenferink A.T., Tibbe A.G., Hiltermann T.J., Groen H.J., van Rijn C.J., Terstappen L.W. (2015). The detection of EpCAM(+) and EpCAM(−) circulating tumor cells. Sci. Rep..

[3] Zhou L., Dicker D.T., Matthew E., El-Deiry W.S., Alpaugh R.K. (2017). Circulating tumor cells: Silent predictors of metastasis. F1000Res.

[4] Kaifi J.T., Li G., Clawson G., Kimchi E.T., Staveley-O'Carroll K.F. (2016). Perioperative circulating tumor cell detection: Current perspectives. Cancer Biol. Ther..

[5] O’Flaherty L., Wikman H., Pantel K. (2017). Biology and clinical significance of circulating tumor cell subpopulations in lung cancer. Transl. Lung Cancer Res..

[6] Kaifi J.T., Kunkel M., Das A., Harouaka R.A., Dicker D.T., Li G., Zhu J., Clawson G.A., Yang Z., Reed M.F. (2015). Circulating tumor cell isolation during resection of colorectal cancer lung and liver metastases: A prospective trial with different detection techniques. Cancer Biol. Ther..

[7] Harouaka R.A., Zhou M.D., Yeh Y.T., Khan W.J., Das A., Liu X., Christ C.C., Dicker D.T., Baney T.S., Kaifi J.T. (2014). Flexible micro spring array device for high-throughput enrichment of viable circulating tumor cells. Clin. Chem..

[8] Clawson G.A., Kimchi E., Patrick S.D., Xin P., Harouaka R., Zheng S., Berg A., Schell T., Staveley-O'Carroll K.F., Neves R.I. (2012). Circulating tumor cells in melanoma patients. PLoS ONE.

[9] Adams D.L., Alpaugh R.K., Tsai S., Tang C.M., Stefansson S. (2016). Multi-Phenotypic subtyping of circulating tumor cells using sequential fluorescent quenching and restaining. Sci. Rep..

[10] Mimeault M., Batra S.K. (2014). Molecular biomarkers of cancer stem/progenitor cells associated with progression, metastases, and treatment resistance of aggressive cancers. Cancer Epidemiol. Biomark. Prev..

[11] Satelli A., Mitra A., Brownlee Z., Xia X., Bellister S., Overman M.J., Kopetz S., Ellis L.M., Meng Q.H., Li S. (2015). Epithelial-mesenchymal transitioned circulating tumor cells capture for detecting tumor progression. Clin. Cancer Res..

[12] Herbst R.S., Baas P., Kim D.W., Felip E., Perez-Gracia J.L., Han J.Y., Molina J., Kim J.H., Arvis C.D., Ahn M.J. (2016). Pembrolizumab versus docetaxel for previously treated, PD-L1-positive, advanced non-small-cell lung cancer (KEYNOTE-010): A randomised controlled trial. Lancet.

[13] Garon E.B., Rizvi N.A., Hui R., Leighl N., Balmanoukian A.S., Eder J.P., Patnaik A., Aggarwal C., Gubens M., Horn L. (2015). Pembrolizumab for the treatment of non-small-cell lung cancer. N. Engl. J. Med..

[14] Guibert N., Delaunay M., Lusque A., Boubekeur N., Rouquette I., Clermont E., Mourlanette J., Gouin S., Dormoy I., Favre G. (2018). PD-L1 expression in circulating tumor cells of advanced non-small cell lung cancer patients treated with nivolumab. Lung Cancer.

[15] Mazel M., Jacot W., Pantel K., Bartkowiak K., Topart D., Cayrefourcq L., Rossille D., Maudelonde T., Fest T., Alix-Panabieres C. (2015). Frequent expression of PD-L1 on circulating breast cancer cells. Mol. Oncol..

[16] Alsuliman A., Colak D., Al-Harazi O., Fitwi H., Tulbah A., Al-Tweigeri T., Al-Alwan M., Ghebeh H. (2015). Bidirectional crosstalk between PD-L1 expression and epithelial to mesenchymal transition: Significance in claudin-low breast cancer cells. Mol. Cancer.

[17] Datar I., Schalper K.A. (2016). Epithelial-Mesenchymal Transition and Immune Evasion during Lung Cancer Progression: The Chicken or the Egg?. Clin. Cancer Res..

[18] Lou Y., Diao L., Cuentas E.R., Denning W.L., Chen L., Fan Y.H., Byers L.A., Wang J., Papadimitrakopoulou V.A., Behrens C. (2016). Epithelial-Mesenchymal Transition Is Associated with a Distinct Tumor Microenvironment Including Elevation of Inflammatory Signals and Multiple Immune Checkpoints in Lung Adenocarcinoma. Clin. Cancer Res..

[19] Chen L., Heymach J.V., Qin F.X., Gibbons D.L. (2015). The mutually regulatory loop of epithelial-mesenchymal transition and immunosuppression in cancer progression. Oncoimmunology.

[20] Chouaib S., Janji B., Tittarelli A., Eggermont A., Thiery J.P. (2014). Tumor plasticity interferes with anti-tumor immunity. Crit. Rev. Immunol..

[21] Chen L., Gibbons D.L., Goswami S., Cortez M.A., Ahn Y.H., Byers L.A., Zhang X., Yi X., Dwyer D., Lin W. (2014). Metastasis is regulated via microRNA-200/ZEB1 axis control of tumour cell PD-L1 expression and intratumoral immunosuppression. Nat. Commun..

[22] Satelli A., Batth I.S., Brownlee Z., Rojas C., Meng Q.H., Kopetz S., Li S. (2016). Potential role of nuclear PD-L1 expression in cell-surface vimentin positive circulating tumor cells as a prognostic marker in cancer patients. Sci. Rep..

[23] Raimondi C., Carpino G., Nicolazzo C., Gradilone A., Gianni W., Gelibter A., Gaudio E., Cortesi E., Gazzaniga P. (2017). PD-L1 and epithelial-mesenchymal transition in circulating tumor cells from non-small cell lung cancer patients: A molecular shield to evade immune system?. Oncoimmunology.

[24] Cronin K.A., Lake A.J., Scott S., Sherman R.L., Noone A.M., Howlader N., Henley S.J., Anderson R.N., Firth A.U., Ma J. (2018). Annual Report to the Nation on the Status of Cancer, part I: National cancer statistics. Cancer.

[25] Adams D.L., Stefansson S., Haudenschild C., Martin S.S., Charpentier M., Chumsri S., Cristofanilli M., Tang C.M., Alpaugh R.K. (2015). Cytometric characterization of circulating tumor cells captured by microfiltration and their correlation to the CellSearch((R)) CTC test. Cytom. A.

[26] Adams D.L., Zhu P., Makarova O.V., Martin S.S., Charpentier M., Chumsri S., Li S., Amstutz P., Tang C.M. (2014). The systematic study of circulating tumor cell isolation using lithographic microfilters. RSC Adv..

[27] Le U.T., Bronsert P., Picardo F., Riethdorf S., Haager B., Rylski B., Czerny M., Beyersdorf F., Wiesemann S., Pantel K. (2018). Intraoperative detection of circulating tumor cells in pulmonary venous blood during metastasectomy for colorectal lung metastases. Sci. Rep..

[28] Rhim A.D., Mirek E.T., Aiello N.M., Maitra A., Bailey J.M., McAllister F., Reichert M., Beatty G.L., Rustgi A.K., Vonderheide R.H. (2012). EMT and dissemination precede pancreatic tumor formation. Cell.

[29] Mitra A., Satelli A., Xia X., Cutrera J., Mishra L., Li S. (2015). Cell-surface Vimentin: A mislocalized protein for isolating csVimentin(+) CD133(-) novel stem-like hepatocellular carcinoma cells expressing EMT markers. Int. J. Cancer.

[30] Lecharpentier A., Vielh P., Perez-Moreno P., Planchard D., Soria J.C., Farace F. (2011). Detection of circulating tumour cells with a hybrid (epithelial/mesenchymal) phenotype in patients with metastatic non-small cell lung cancer. Br. J. Cancer.

[31] Chakravarti N., Prieto V.G. (2015). Predictive factors of activity of anti-programmed death-1/programmed death ligand-1 drugs: Immunohistochemistry analysis. Transl. Lung. Cancer Res..

[32] Gandini S., Massi D., Mandala M. (2016). PD-L1 expression in cancer patients receiving anti PD-1/PD-L1 antibodies: A systematic review and meta-analysis. Crit. Rev. Oncol. Hematol..

[33] Yue C., Jiang Y., Li P., Wang Y., Xue J., Li N., Li D., Wang R., Dang Y., Hu Z. (2018). Dynamic change of PD-L1 expression on circulating tumor cells in advanced solid tumor patients undergoing PD-1 blockade therapy. Oncoimmunology.

[34] Nicolazzo C., Raimondi C., Mancini M., Caponnetto S., Gradilone A., Gandini O., Mastromartino M., Del Bene G., Prete A., Longo F. (2016). Monitoring PD-L1 positive circulating tumor cells in non-small cell lung cancer patients treated with the PD-1 inhibitor Nivolumab. Sci. Rep..

[35] Kim S., Koh J., Kim M.Y., Kwon D., Go H., Kim Y.A., Jeon Y.K., Chung D.H. (2016). PD-L1 expression is associated with epithelial-to-mesenchymal transition in adenocarcinoma of the lung. Hum. Pathol..

[36] Ghebeh H., Lehe C., Barhoush E., Al-Romaih K., Tulbah A., Al-Alwan M., Hendrayani S.F., Manogaran P., Alaiya A., Al-Tweigeri T. (2010). Doxorubicin downregulates cell surface B7-H1 expression and upregulates its nuclear expression in breast cancer cells: Role of B7-H1 as an anti-apoptotic molecule. Breast Cancer Res..

[37] Luo W., Fang W., Li S., Yao K. (2012). Aberrant expression of nuclear vimentin and related epithelial-mesenchymal transition markers in nasopharyngeal carcinoma. Int. J. Cancer.

[38] Luo W.R., Wu A.B., Fang W.Y., Li S.Y., Yao K.T. (2012). Nuclear expression of N-cadherin correlates with poor prognosis of nasopharyngeal carcinoma. Histopathology.

[39] Azuma T., Yao S., Zhu G., Flies A.S., Flies S.J., Chen L. (2008). B7-H1 is a ubiquitous antiapoptotic receptor on cancer cells. Blood.

[40] Adams D.L., Adams D.K., Stefansson S., Haudenschild C., Martin S.S., Charpentier M., Chumsri S., Cristofanilli M., Tang C.M., Alpaugh R.K. (2016). Mitosis in circulating tumor cells stratifies highly aggressive breast carcinomas. Breast Cancer Res..

[41] Adams D.L., Martin S.S., Alpaugh R.K., Charpentier M., Tsai S., Bergan R.C., Ogden I.M., Catalona W., Chumsri S., Tang C.M. (2014). Circulating giant macrophages as a potential biomarker of solid tumors. Proc. Natl. Acad. Sci. USA.

[42] Tsuchiya B., Sato Y., Kameya T., Okayasu I., Mukai K. (2006). Differential expression of N-cadherin and E-cadherin in normal human tissues. Arch. Histol. Cytol..

[43] Pepe M.S., Feng Z., Janes H., Bossuyt P.M., Potter J.D. (2008). Pivotal evaluation of the accuracy of a biomarker used for classification or prediction: Standards for study design. J. Natl. Cancer Inst..

